# Delayed postoperative radiotherapy increases the incidence of radiographic local tumor progression before radiotherapy and leads to poor prognosis in spinal metastases

**DOI:** 10.1186/s13014-020-01740-y

**Published:** 2021-01-22

**Authors:** Yining Gong, Hongqing Zhuang, Shan Chong, Qianyu Shi, Feng Wei, Zhongjun Liu, Hanqiang Ouyang, Xiaoguang Liu, Liang Jiang

**Affiliations:** 1 Department of Orthopedics, Peking University Third Hospital, 49 North Garden Road, Haidian District, Beijing, 100191 China; 2 Health Science Center, Peking University, Beijing, China; 3 Department of Radiation Oncology, Peking University Third Hospital, Beijing, China; 4 The Institute of Traumatology and Orthopaedics, Beijing Jishuitan Hospital, Beijing, China

**Keywords:** Spinal metastases, Postoperative radiotherapy, Time interval, Prognosis, Local control, Overall survival

## Abstract

**Background:**

Most previous studies focused on the minimum interval between surgery and radiotherapy in spinal metastases, leaving the maximum interval under-investigated. However, in real world, limited radiotherapist and equipment cannot meet the needs of a large patient population to obtain timely radiotherapy after the index spine surgery in developing countries. This study aimed to estimate the clinical risks of delayed radiotherapy after surgery in patients with spinal metastases in developing country.

**Methods:**

Data from 89 patients who underwent surgery and postoperative radiotherapy at a single site in a developing country were retrospectively reviewed. Patients were divided into the progression before radiotherapy (PBR) and no progression before radiotherapy (NPBR) groups. Kaplan–Meier analysis and log-rank tests were used to compare the local control (LC) and overall survival (OS) between groups.

**Results:**

Within 1 month after surgery, only 20.2% of patients underwent radiotherapy. Risk of local progression before radiotherapy at 1, 3, and 6 months was 1.2%, 24.1%, and 45.1%, respectively. The LC rate at 1 year was lower in the PBR group than in the NPBR group (53.3% vs. 76.3%, *P* = 0.040). The OS rate at 1 year was 61.9% and 79.6% in the PBR and NPBR groups, respectively (*P* = 0.001). The Karnofsky performance status significantly improved only in the NPBR group (52.5 ± 17.6 vs. 66.8 ± 26.3, *P* < 0.001). The sphincter dysfunction significantly improved in the NPBR group (0.3 ± 0.5 vs. 0.1 ± 0.3, *P* = 0.007) but it tended to be deteriorated in the PBR group (0.1 ± 0.4 vs. 0.3 ± 0.5, *P* = 0.500).

**Conclusions:**

In real world, about 80% of patients had delayed radiotherapy 1 month after spine surgery for metastases in our developing country. Patients had a higher risk for radiographic local progression before radiotherapy and poorer LC, OS, and quality of life as time to radiotherapy increased.

## Background

The spine is the most common site of skeletal metastases and it is estimated that the incidence of spinal metastases is 30%-50% in patients with cancer [1, 2]. Spinal metastases may involve epidural spinal cord compression (ESCC) with or without bone destruction, leading to intractable pain, pathologic fractures, or spinal cord or nerve root compression, all of which could significantly impact patients’ quality of life [3]. In selected patients, radiotherapy alone can be an important option, in particular with stereotactic technique [4, 5]. However, surgical treatment is required before radiotherapy when there is high-grade ESCC or spinal instability [6]. Recent advancements in treatment include sufficient epidural decompression (such as separation surgery) followed by intensity-modulated radiotherapy (IMRT) or stereotactic body radiotherapy (SBRT) to achieve durable local control (LC) and lower the incidence of marginal failures [7–9].

To avoid postoperative tumor progression, radiotherapy is carried out as soon as possible after the index spine surgery. Since the radiation might impair the postoperative healing process, an interval of at least 1 or 2 weeks between surgery and radiotherapy is recommended [10–13]. Most previous studies focused on the minimum interval between surgery and radiotherapy, leaving the maximum interval under-investigated. Delayed postoperative radiotherapy may be associated with the risk of residual tumor recurrence or reduced sensitivity to radiation [10]. However, in developing countries, limited radiotherapist and equipment cannot meet the needs of a large patient population to obtain timely radiotherapy after the index spine surgery. Patients with spine metastases treated at the present study site are typically suggested to undergo radiotherapy 3–4 weeks after the index spine surgery. However, there are several reasons, such as patient weakness due to the index surgery or poor general health, preference for alternative treatment, and overburdened radiotherapy workforce, due to which some patients do not undergo postoperative radiotherapy during the recommended timeframe.

Missing the optimal timeframe for postoperative radiation might result in a poor prognosis and even recurrence of spinal cord compression. Therefore, this study aimed to calculate the risk of radiographic local progression before radiotherapy in patients with spinal metastases according to the time interval between index surgery and radiation treatment and describe the influence of delayed radiotherapy on patient prognosis.

## Methods

### Study design and participants

**Fig. 1:**
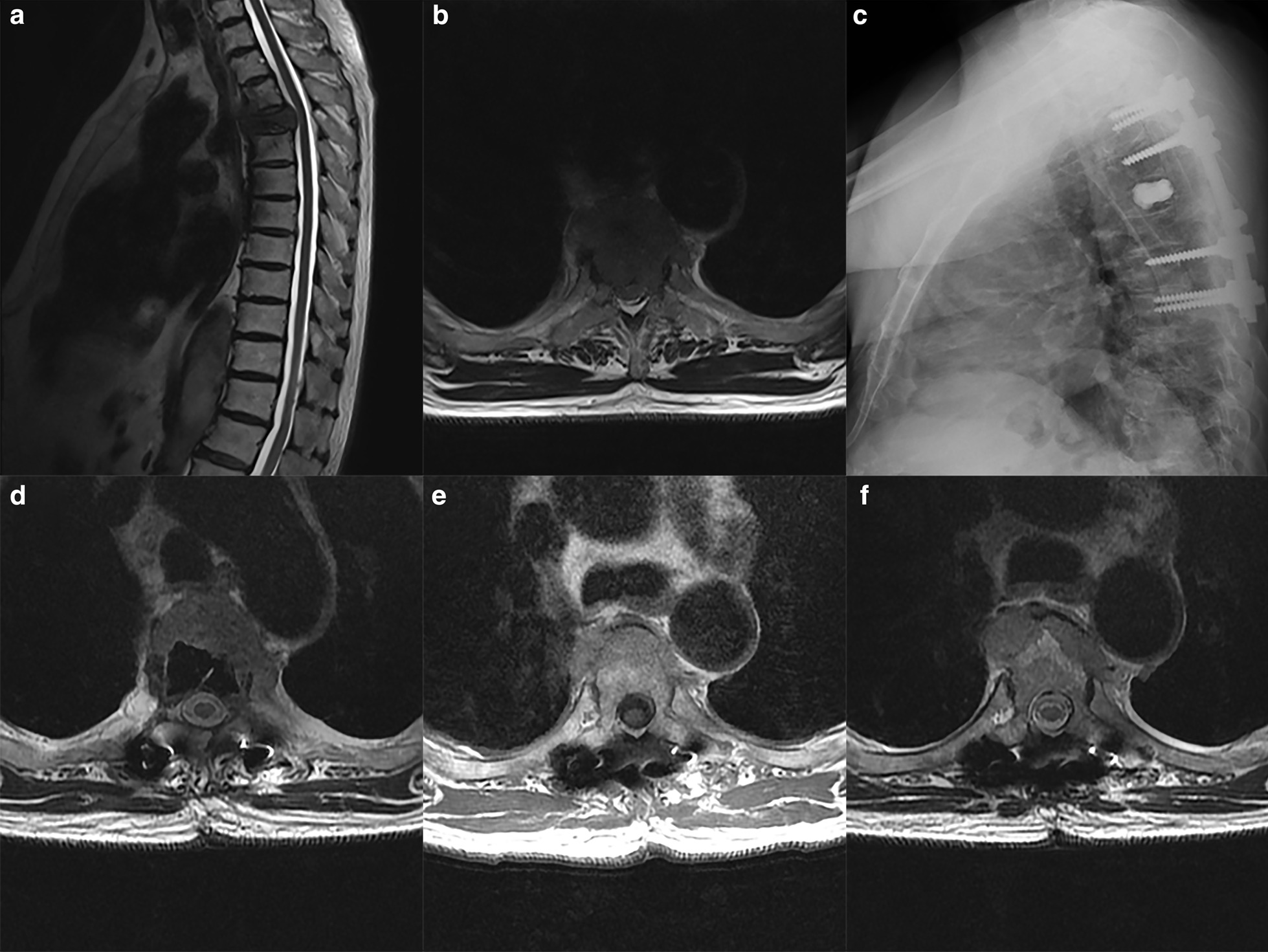
Imaging of patients in progression before radiotherapy group (PBR). A 77-year-old man with T4 metastatic lung cancer experienced severe back pain and weakness for 2 months. **a**, **b** Sagittal and axial T2-weighted magnetic resonance imaging (MRI) before surgery showed a lesion in T4 with high-grade epidural spinal cord compression (ESCC). **c** Separation surgery was performed and postoperative radiograph indicated well fixation. **d** Postoperative axial T2-weighted MRI showing complete decompression of the spinal cord. Compared with postoperative image (**e**, 5 days after surgery), axial T2-weighted MRI showed local progression before radiotherapy (**f**, 50 days after surgery). The two images displayed the slices at the same level in T5 superior endplate

This retrospective study was conducted in accordance with the Strengthening the Reporting of Observational Studies in Epidemiology (STROBE) Statement [14] and the institutional review board approved the study design. Data of patients who underwent surgical treatment with postoperative radiotherapy for spinal metastases in a high-volume tertiary teaching hospital in China from October 2010 to March 2020 were collected. Inclusion criteria were as follows: (1) confirmed spinal metastases, (2) both surgical treatment and postoperative radiotherapy were performed at the study site, (3) separation surgery, decompression surgery (laminectomy), and vertebrectomy, (4) underwent IMRT or SBRT, and (5) time interval between surgical treatment and postoperative radiotherapy less than 9 months. The exclusion criteria were as follows: (1) surgical treatment or radiotherapy not performed at study-site, (2) vertebroplasty, occipitocervical fusion without tumor resection, and total vertebra resection; (3) radioactive seed implantations, and (4) unavailable pre-radiotherapy magnetic resonance imaging (MRI) data. The follow-up was a minimum of 6 months after radiotherapy or until the death of a patient. MRI was performed before radiotherapy to identify any local tumor progression (Fig. 1). MRI data were reviewed by a radiologist and an orthopedic surgeon. Any discrepancies between the reviewers were resolved by another senior surgeon. According to the MRI data, the patients were divided into two groups, namely, local progression before radiotherapy (PBR) and no progression before radiotherapy (NPBR).

### Data collection

Patient and tumor characteristics were collected from electronic medical records and our specialized database, including age, sex, tumor histology, location, ESCC grade [15], spinal instability neoplastic score (SINS) [16], Tomita score [17], revised Tokuhashi score [18], radiotherapy method, overall survival (OS), and LC.

Patients underwent regular clinical and radiography follow-up. The primary outcomes included OS and LC. Based on the 2015 Spine Response Assessment in the Neuro-Oncology Group report, LC was defined as a lack of tumor progression within the treated volume [19]. Quality of life parameters were collected both preoperatively and during follow-up and included the numeric rating scale (NRS) score, Karnofsky performance status (KPS) score, mobility (bedridden, wheelchair, double-crutches, single-crutches, or walking independently were assigned 0–4 points, respectively), and sphincter dysfunction (0 or 1 for negative or positive).

### Statistical analyses

Statistical analysis was performed using SPSS version 24.0 (IBM, Armonk, NY, USA). Data were summarized as number of patients (percentage), mean ± standard deviation and median (range), accordingly. The Kaplan–Meier analysis and log-rank test were used to analyze and compare the LC and OS between groups. Comparisons of NRS, KPS, mobility, and sphincter dysfunction between pre-surgery and at follow-up were performed using the Wilcoxon test. Comparisons of parameters between the PBR and NPBR groups were performed using the Mann–Whitney U test. A *P*-value of less than 0.05 was considered statistically significant.

## Results

### Patient and tumor characteristics

A total of 89 successive patients were included in the study. The average age at surgery was 54.6 ± 11.6 years and the proportion of males was slightly higher than females. The most common primary tumor type was renal cancer (22.5%, 20/89), followed by lung (21.3%, 19/89) and breast cancer (14.6%, 13/89). The most common sites of spinal metastases were the thoracic (40.4%, 36/89) segments. The ESCC grade before surgery was 3 in more than half of the patients (58.4%, 52/89), while patients with grade 2 accounted for 32.6% (29/89). Operation types included separation surgery (57.3%, 51/89), decompression surgery (25.8%, 23/89), and vertebrectomy (16.9%, 15/89). The majority (71.9%, 64/89) of patients underwent post-surgical SBRT, while others were treated with IMRT. There was no significant difference between NPBR and PBR in the above parameters (Table 1). Both in NPBR and PBR, median prescription dose/ number of fractions of SBRT and IMRT were 35 Gy/5 fractions and 30 Gy/10 fractions, respectively. Only three patients were lost to follow-up, resulting in a 96.6% (86/89) of the follow-up rate. The average follow-up time after radiotherapy was 15.8 ± 12.8 months.

**Table 1:** Patient and tumor characteristics

Characteristic	NPBR^a^	PBR^a^	*P*-value
Age (years)	53.7 ± 11.5	59.4 ± 11.8	0.082
Sex			0.386
Male	43 (57.3%)	10 (71.4%)	
Female	32 (42.7%)	4 (28.6%)	
Tumor histology			0.404
Renal	17 (22.7%)	3 (21.4%)	
Lung	16 (21.3%)	3 (21.4%)	
Breast	13 (17.3%)	0 (0.0%)	
Thyroid	5 (6.7%)	3 (21.4%)	
Hepatocellular	3 (4.0%)	1 (7.1%)	
Colorectal	4 (5.3%)	1 (7.1%)	
Others	17 (22.7%)	3 (21.4%)	
Location			0.341
Cervical	20 (26.7%)	7 (50.0%)	
Thoracic	31 (41.3%)	5 (35.7%)	
Lumbar	15 (20.0%)	1 (7.1%)	
Cervical and thoracic	3 (4.0%)	1 (7.1%)	
Thoracic and lumbar	6 (8.0%)	0 (0.0%)	
ESCC grade			0.244
0	1 (1.3%)	0 (0.0%)	
1b	1 (1.3%)	0 (0.0%)	
1c	5 (6.7%)	1 (7.1%)	
2	21 (28.0%)	8 (57.1%)	
3	47 (62.7%)	5 (35.7%)	
SINS	11.1 ± 2.2	10.4 ± 2.2	0.247
Tomita score	5.2 ± 2.0	4.9 ± 1.9	0.657
Tokuhashi score	8.4 ± 2.8	9.0 ± 2.9	0.453
Operation types			0.436
Separation surgery	44 (58.7%)	7 (50.0%)	
Decompression surgery	20 (26.7%)	3 (21.4%)	
Vertebrectomy	11 (14.7%)	4 (28.6%)	
Radiotherapy methods			1.000
IMRT	21 (28.0%)	4 (28.6%)	
SBRT	54 (72.0%)	10 (71.4%)	

ESCC, epidural spinal cord compression; IMRT, intensity-modulated radiotherapy; NPBR, no progression before radiotherapy; PBR, progression before radiotherapy; SBRT, stereotactic body radiotherapy; SINS, spine instability neoplastic score

^a^Values are expressed as mean ± standard deviation or number of patients (percentage in each group)

### Radiographic local progression before radiotherapy

**Fig. 2:**
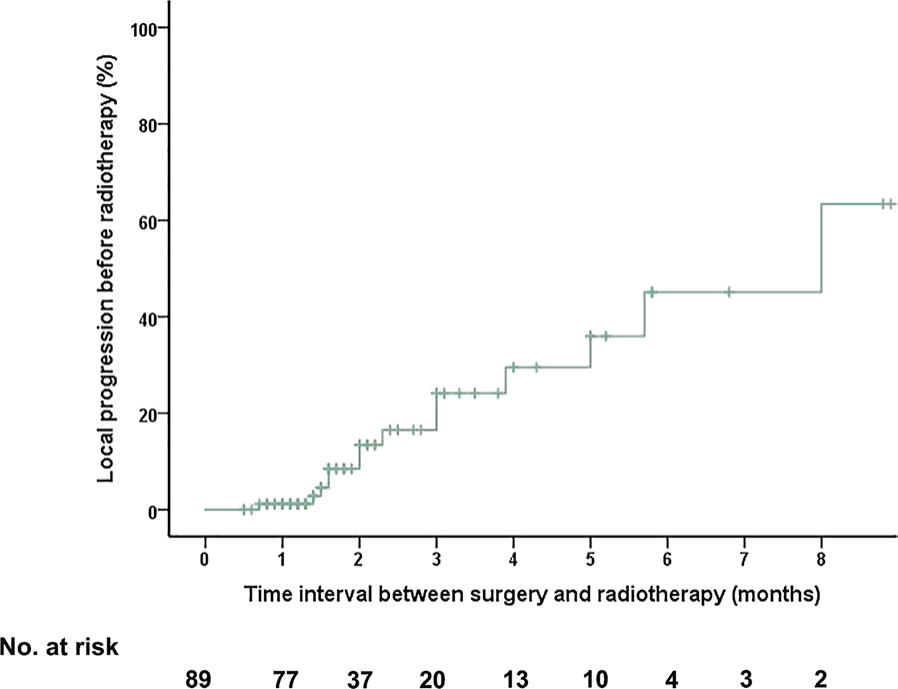
Radiographic local tumor progression before radiotherapy in all patients

**Fig. 3:**
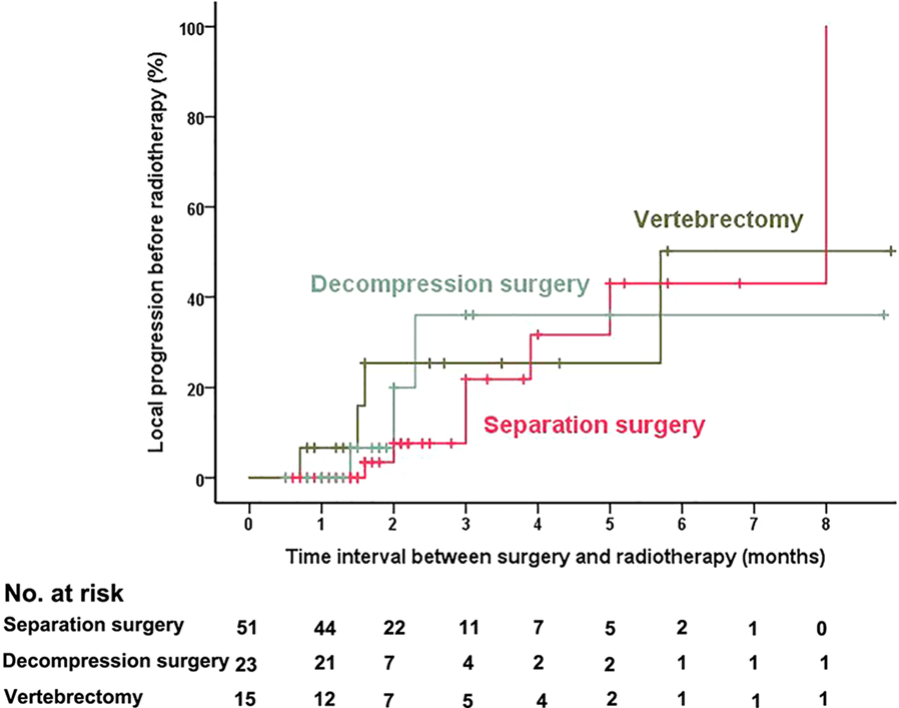
Radiographic local tumor progression before radiotherapy according to operation types

The median time interval (TI) between surgery and radiotherapy was 1.6 months (range: 0.5 to 8.9). Altogether, 18 (20.2%), 53 (59.6%), 14 (15.7%), and 4 (4.5%) patients underwent radiotherapy after surgery within 1, 1–3, 3–6 and 6–9 months, respectively. A total of 15.7% (14/89) patients developed radiographic local progression before radiotherapy, and the estimated risk for radiographic local progression before radiotherapy at 1, 3, and 6 months was 1.2%, 24.1%, and 45.1%, respectively (Fig. 2). Only one patient (gastric cancer, vertebrectomy, TI = 0.7 month) developed radiographic local progression within 1 month. The percentage of patients with local progression before radiotherapy was 13.7% (7/51) in the separation surgery group, 13.0% (3/23) in the decompression surgery group, and 26.7% (4/15) in the vertebrectomy group. However, there was no significant difference according to the Kaplan–Meier analysis among different operation types (*P* = 0.884) (Fig. 3).

### Prognosis of patients between the PBR and NPBR groups

**Fig. 4:**
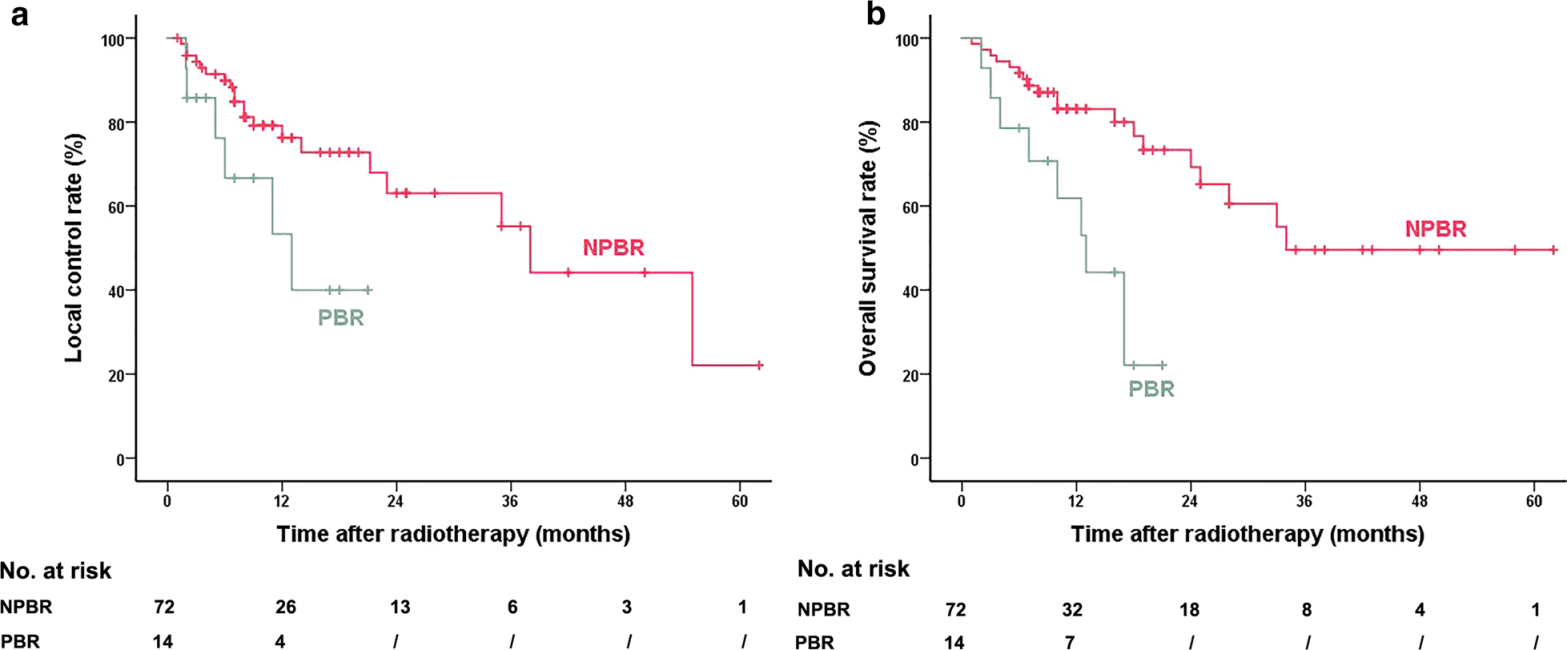
Local control and overall survival after radiotherapy in the two groups. Estimated rates of LC (**a**) and OS (**b**). LC, local control; NPBR, no progression before radiotherapy group; OS, overall survival; PBR, progression before radiotherapy group

According to Kaplan–Meier analysis, the estimated overall LC rate at 1 year after radiotherapy was 72.8%. The estimated LC rate at 1 year was 53.3% in the PBR group, which was significantly lower than that in the NPBR group (76.3%, *P* = 0.040). The estimated overall OS rate at 1 year after radiotherapy was 79.6%. In the PBR group, the estimated OS rate at 1 year was 61.9%, while that in the NPBR group was significantly higher at 83.1% (*P* = 0.001) (Fig. 4).

For other patient outcomes, there were significant differences in preoperative- and follow-up NRS scores for pain in both groups (NPBR group: 6.8 ± 2.2 vs. 3.2 ± 2.6, *P* < 0.001; PBR group: 6.5 ± 2.0 vs. 4.2 ± 3.1, *P* = 0.012). Between the two groups at the last follow-up, the NPBR vs. PBR group NRS scores tended to be lower but without a significant difference 3.2 ± 2.6 vs. 4.2 ± 3.1, *P* = 0.253). According to the KPS, significant improvement between preoperative and follow-up scores occurred only in the NPBR group (52.5 ± 17.6 vs. 66.8 ± 26.3, *P* < 0.001). Mobility was improved in the NPBR group (2.6 ± 1.7 vs. 3.0 ± 1.6, *P* = 0.086) with borderline significant difference, but it tended to be deteriorated in the PBR group (2.9 ± 1.7 vs. 2.5 ± 1.8, *P* = 0.531). Besides, the mobility time after radiotherapy was longer in the NPBR group with borderline significant difference (14.1 ± 12.8 vs. 8.3 ± 7.4 months, *P* = 0.086). The sphincter dysfunction was improved in the NPBR group (0.3 ± 0.5 vs. 0.1 ± 0.3, *P* = 0.007) but it tended to be deteriorated in the PBR group (0.1 ± 0.4 vs. 0.3 ± 0.5, *P* = 0.500) (Table 2)**.**

**Table 2:** Patient-reported outcomes

Outcomes	NPBR^a^	PBR^a^	*P*-value^b^
NRS			
Preoperative	6.8 ± 2.2	6.5 ± 2.0	0.469
Follow-up	3.2 ± 2.6	4.2 ± 3.1	0.253
* P* value^c^	< 0.001	0.012	
KPS			
Preoperative	52.5 ± 17.6	55.0 ± 19.9	0.559
Follow-up	66.8 ± 26.3	55.7 ± 26.8	0.115
* P* value^c^	< 0.001	0.879	
Mobility			
Preoperative	2.6 ± 1.7	2.9 ± 1.7	0.532
Follow-up	3.0 ± 1.6	2.5 ± 1.8	0.194
* P* value^c^	0.086	0.531	
Mobility time (months)			
Follow-up	14.1 ± 12.8	8.3 ± 7.4	0.086
Sphincter dysfunction			
Preoperative	0.3 ± 0.5	0.1 ± 0.4	0.343
Follow-up	0.1 ± 0.3	0.3 ± 0.5	0.213
* P* value^c^	0.007	0.500	

KPS, Karnofsky performance status; NPBR, no progression before radiotherapy; NRS, numeric rating scale; PBR, progression before radiotherapy

^a^Values are expressed as mean ± standard deviation

^b^Comparison between the two groups

^c^Comparison of preoperative- and follow-up values

### Complications

Surgery-related complications included wound infection (five patients), implant failure (three patients), transient neurologic deficit (two patients), urinary tract infection (two patients), leakage of cerebrospinal fluid (one patients), dyspnea for edema of the posterior pharyngeal wall after cervical surgery (one patient), respiratory and heart failure (one patient), pneumonia and hydrothorax (one patient), and transient delirium (one patient).

Radiotherapy-related complications included vertebral compression fracture (one patient, TI = 1.0 month, NPBR), radiation radiculopathy (one patient, TI = 2.5 months, NPBR), and bacteremia (one patient, TI = 5.7 months, PBR).

## Discussion

In the present study, the prognosis was analyzed in patients who underwent radiotherapy after surgery for spinal metastases at a single-site in a developing country. Overall, about 15.7% of patients developed radiographic local progression before radiotherapy. The percentage of patients with local progression was increased as the interval between surgery and radiotherapy increased. Results also showed that post-radiotherapy LC and OS rates were poorer in patients with PBR, the KPS scores, mobility and sphincter dysfunction was improved only in NPBR patients at follow-up.

An evidence-based recommended time interval between surgery and radiotherapy for the management of spinal metastases has been previously discussed, but the conclusions associated with it have been unclear. This is partly due to the focus being on the minimum interval, leaving the maximum interval under-investigated. For example, Itshayek et al. suggested that the key factor that limited the minimum time interval was wound healing and bone fusion [11, 12]. Since the radiation impairs these processes, especially in the first week post-surgery, they recommended at least a 1-week interval between surgery and radiotherapy. Lee et al. investigated both radiation oncologists’ and spine surgeons’ opinions and concluded that the time between treatments should be a minimum of 2 weeks [13]. For minimally invasive surgery, radiotherapy can be started within 2 to 3 days after surgery [20]. The present study adds new findings to the existing literature by focusing on the maximal interval in cases of delayed radiotherapy.

Radiotherapy that is performed more than 1 month after the surgery was usually considered delayed radiotherapy in our consensus. One patient with gastric cancer had local progression within the 1-month interval between surgery and radiotherapy resulting in a risk of radiographic local progression before radiotherapy being 1.2%. However, the estimated risk of local progression increased over time, equaling 24.1% at 3 months. Based on these results, radiotherapy after surgery should occur at least within 1 month. However, more clinical evidence is needed to confirm the recommendation.

In the present study, the patient prognosis was promising when radiotherapy was conducted prior to local progression and previous studies have verified the efficacy of surgery and postoperative radiosurgery [6, 21, 22]. Herein, the estimated risk of LC at 1 year in patients without pre-radiotherapy progression was 76.3%. Further, the PBR and NPBR groups were compared to explore the effect of progression before radiotherapy on the prognosis in patients after radiotherapy. Patients with PBR had worse LC, indicating that postponement of radiotherapy led to a worse prognosis. Patients with NPBR also had a longer OS time in the present study. As OS was mainly determined by the primary tumor histology and major organ metastases [23, 24], tumors with local progression before radiotherapy could probably be more malignant in histology and thus more likely to metastasize viscerally, reducing estimated survival time.

Pain relief is considered one of the important aspects in the management of patients with bone metastases [25]. Patients from both groups in the present study had significant pain relief after the treatment. However, in terms of KPS scores and mobility, they were improved only in the patients with NPBR. Besides, the mobility time after radiotherapy was longer in the NPBR group with borderline significant difference. The difference between groups was significant according to the sphincter dysfunction where it in patients with NPBR was improved significantly after radiotherapy while in those with PBR, it had deteriorated but without significant difference. These findings are related to the quality of life in patients and emphasize the importance of timely radiotherapy.

Short TI might impair the postoperative healing process. However, in the present study, wound infection in five patients was developed before postoperative radiation. It was less related to radiation therapy. No wound complication was seen after radiotherapy. Although it seemed that the patients with radiotherapy-related complications had longer TI and TI time in all of them was ≥ 1 month, it was difficult to demonstrate a clear correlation between the complications and the TI.

There are several limitations to the current study to consider. The retrospective study design does not show causality and is, therefore, less powerful to address the issue of delayed radiotherapy. However, a prospective study is not ethically feasible. In addition, tumors with different histology grow at different rates, which may lead to different risk levels associated with delayed treatment. Larger sample size would be needed to conduct this type of the stratified analysis.

## Conclusions

In conclusion, in real world, about 80% of the patients had delayed radiotherapy 1 month after the spine surgery for metastases in our developing country, even in tertiary teaching hospital. Patients had a higher risk for radiographic local progression before radiotherapy and poorer LC, OS, and quality of life as the time to radiotherapy increased.

## Data Availability

The datasets used and/or analysed during the current study are available from the corresponding author on reasonable request.

## Abbreviations

cEBRT: Conventional external-beam radiotherapy
ESCC: Epidural spinal cord compression
IMRT: Intensity-modulated radiotherapy
KPS: Karnofsky performance status
LC: Local control
MRI: Magnetic resonance imaging
NPBR: No progression before radiotherapy
NRS: Numeric rating scale
OS: Overall survival
PBR: Progression before radiotherapy
SBRT: Stereotactic body radiotherapy
SINS: Spinal instability neoplastic score
STROBE: Strengthening the Reporting of Observational Studies in Epidemiology
TI: Time interval
